# Augmented Reality for extremity hemorrhage training: a usability study

**DOI:** 10.3389/fdgth.2024.1479544

**Published:** 2025-01-06

**Authors:** Krishant Tharun, Alberto Drogo, Carmine Tommaso Recchiuto, Serena Ricci

**Affiliations:** ^1^ Department of Informatics, Bioengineering, Robotics and Systems Engineering, University of Genoa, Genoa, Italy; ^2^ Department of Pathophysiology and Transplantation, University of Milan, Milan, Italy; ^3^ Simulation and Advanced Education Center - SimAv, University of Genoa, Genoa, Italy

**Keywords:** augmented reality, simulation-based training, anti-hemorrhage devices, massive limb bleeding, hemorrhage management, healthcare simulation

## Abstract

**Introduction:**

Limb massive hemorrhage is the first cause of potentially preventable death in trauma. Its prompt and proper management is crucial to increase the survival rate. To handle a massive hemorrhage, it is important to train people without medical background, who might be the first responders in an emergency. Among the possible ways to train lay rescuers, healthcare simulation allows to practice in a safe and controlled environment. In particular, immersive technologies such as Virtual Reality (VR) and Augmented Reality (AR) give the possibility to provide real time feedback and present a realistic and engaging scenario, even though they often lack personalization.

**Methods:**

This work aims to overcome the above-mentioned limitation, by presenting the design, development and usability test of an AR application to train non-experienced users on the use of antihemorrhagic devices. The application combines a Microsoft Hololens2 headset, with an AR application developed in Unity Game Engine. It includes a training scenario with a multimodal interactive system made of visual and audio cues, that would adapt to user's learning pace and feedback preference.

**Results:**

Usability tests on 20 subjects demonstrated that the system is well tolerated in terms of discomfort and workload. Also, the system has been high rated for usability, user experience, immersion and sense of presence.

**Discussion:**

These preliminary results suggest that the combination of AR with multimodal cues can be a promising tool to improve hemorrhage management training, particularly for unexperienced users. In the future, the proposed application might increase the number of people who know how to use an anti-hemorrhagic device.

## 1 Introduction

Hemorrhage control is a crucial aspect in emergency situations, as massive bleeding is the first cause of potentially preventable death in trauma (1). Indeed, proper management of hemorrhage is key in preventing excessive blood loss, which can be life-threatening (2). In the case of massive hemorrhage, which can be identified looking for a continuous bleeding, a large-volume bleeding and/or pooling of blood, different strategies and techniques to stop the bleeding can be used (3). These include methods such as applying constant and direct pressure to the wound, using hemostatic agents or hemostatic dressing, and applying tourniquets (i.e., anti-hemorrhagic devices) if the bleeding originates from the limbs (3). Traditionally, tourniquets have been used in military settings and by Emergency Medical Services; however, there is strong evidence in the literature supporting their use by non-medical personnel (4), as a proper use of tourniquets is associated with increased survival in trauma patients (5). Nevertheless, it is important to be aware of the potential risks involved with the improper use of commercial tourniquets such as tissue damage, muscle injury and risk of worsen bleeding if the pressure is insufficient (6). During an emergency anyone present at the scene could be the first responder; therefore, it is crucial for individuals without medical background to learn how to properly and promptly manage a massive hemorrhage prior to professional medical help (7). Possible errors that inexperienced users can make include putting the tourniquet in an incorrect position, applying wrongly, or tightening the device too weakly (8). For all these reasons, proper training in this context is of the utmost importance. Indeed, experiments with participants who completed hemorrhage control training show promising improvement in confidence and proficiency in using hemorrhage control techniques and placing tourniquets (2). With that in mind, in 2015, the American College of Surgeons (ACS; Chicago, Illinois USA) launched the Stop the Bleed campaign which has trained nearly 4 million people worldwide (9). Despite the unquestionable value of Stop the Bleed courses, retention of the medical skills in absence of refresh courses or constant practice retrieves significantly (10). Therefore, a training setting that can be achieved anytime and allows multiple repetitions, also without the presence of an instructor, could be helpful to retain skills and, also, train more people (11).

Among the possible ways to train lay rescuers, there is simulation which allows to practice in a realistic but riskless and controlled environment (10). Immersive technologies such as Virtual Reality (VR) and Augmented Reality (AR) are gaining interest among the medical education community given their possibility of providing real time feedback and presenting realistic and engaging scenarios. In particular, AR, that enhances the real world with computer-generated content, can facilitate effective communication and skill transfer, which is particularly relevant in medical training scenarios (12, 13). However, one of the current limitations of immersive solutions for medical training, lies in the lack of personalization. Currently, the difference between instructor-guided courses and self-learning through VR/AR application is the possibility to tailor the courses based on the learner’s needs, difficulties and abilities (11). In other words, if the trainees are inclined to learn through practical examples and active participation, the instructor might consider expanding the hands-on activities; if they prefer diagrams or rather real-life examples, he/she can adjust the way the content is delivered. Indeed, as instructors adjust the training according to the trainee, the same way VR/AR applications should be adapted to the end users, to guarantee engaging and challenging experiences which promote learning (11). At the current stage, VR/AR-based tools deliver a predefined content which does not consider the specific user’s needs and is not self-adjustable in terms of how to provide the content.

The goals of this study were to (i) develop a novel AR application to train non-experienced users on how to manage a limb massive hemorrhage, which combines AR with multimodal cues; (ii) assess its usability and workload among potential and users. We try to overcome the limitations related to the lack of personalized AR-based trainings by developing a system which provides different type of cues. During the training, users receives cues, in the form of images, icons, text, and oral instructions; they can decide what to look at and how to interact with the system. We hypothesize that the system will be well tolerated and evaluated by end users, due to the combination of different feedback which take into account trainees’ learning pace and feedback preference. For the same reason, we foresee similar completion timings among trainees, who could rely on different tyepes of feedback. Finally, we expect an overall low workload, despite many users will interact with AR for the first time. Indeed, we have included a structured familiarization phase before the training, which should reduce the training workload by showing trainees how to use the AR application.

## 2 Related work

The use of immersive technologies for medical training is a relatively new field, that has been growing over the past 10 years (14). The healthcare simulation community agrees that there is a need for research studies on the design, development and use of immersive technologies for medical training, to better understand their potentialities, limitations and how to use them in a standardized way to promote skills learning and retention (15). Regarding the different technologies used, VR is more common than AR. This might be because VR headsets are generally cheaper than AR ones. In addition, there is often a need to isolate the trainee using fully virtual environments. This ultimately leads to fewer studies investigating the potentialities of AR. However, AR promotes learning by providing realistic and immersive experiences and it is generally appreciated by both trainees and experts (16). Literature studies report that AR-based learning can enhance the medical student experience and translate it into improved learning outcomes, including enhanced theoretical knowledge, practical skills, non-technical skills, and competence (17). This is also confirmed by the work of (18), which reports that medical training done by using immersive technologies can improve skills and decision making in medical scenarios.

Currently, several AR applications for medical training have been developed in different fields, such as anatomy (19, 20), and surgery (21). In all these contexts, the general feedback from trainees and experts in implementing AR in medical education has been highly positive. AR applications range from smartphone applications (i.e., video see through) up to more complex optical see through ones, that use semi-transparent AR glasses to combine the virtual content with the real view of the world (11). As an example, an AR application developed to facilitate learning and training of electrocardiography (ECG) chest lead placement via smartphones has been demonstrated to improve accuracy and learning efficiency (22). Other applications have been developed in the field of basic and advanced life support (23–26), even though efficacy studies are still limited (11). Furthermore, promising applications of AR include the training of procedural knowledge and the use of medical machines. Speaking about the simulations of virtual patients and assessment methods, the work of (27, 28) provided insights about new trends in computer-aided teaching and assessment methods, with a special focus on AR and VR techniques for the assessment of students competency. Finally, Maleszka et al. (29) developed an AR application prototype that displays checklists for the anesthesia induction process, designed to be used in combination with existing anesthesia simulations. This prototype allows the user to set different parameters and provides information on how to use the device.

In the specific field of trauma management, there is general agreement that current disaster training for medical first-responders is inadequate, as it lack realism (30). In this context, immersive technologies can enhance preparedness among medical first responders (30). Koutitas et al. (31) proposed an AR/VR-based training framework for first responders; the study showed that this training methodology improved both the accuracy and the speed of first responders (31). Nevertheless, AR/VR-based systems for trauma management training are limited to few commercial solutions, that however have not been evaluated by the scientific community yet, specially in their capability to measure the efficacy of maneuvers. Most importantly, no current available AR or VR tools provide personalized and self-adjustable programs, which are at the basis of any well-conducted instructor-based simulation.

## 3 Materials and methods

**Figure 1:**
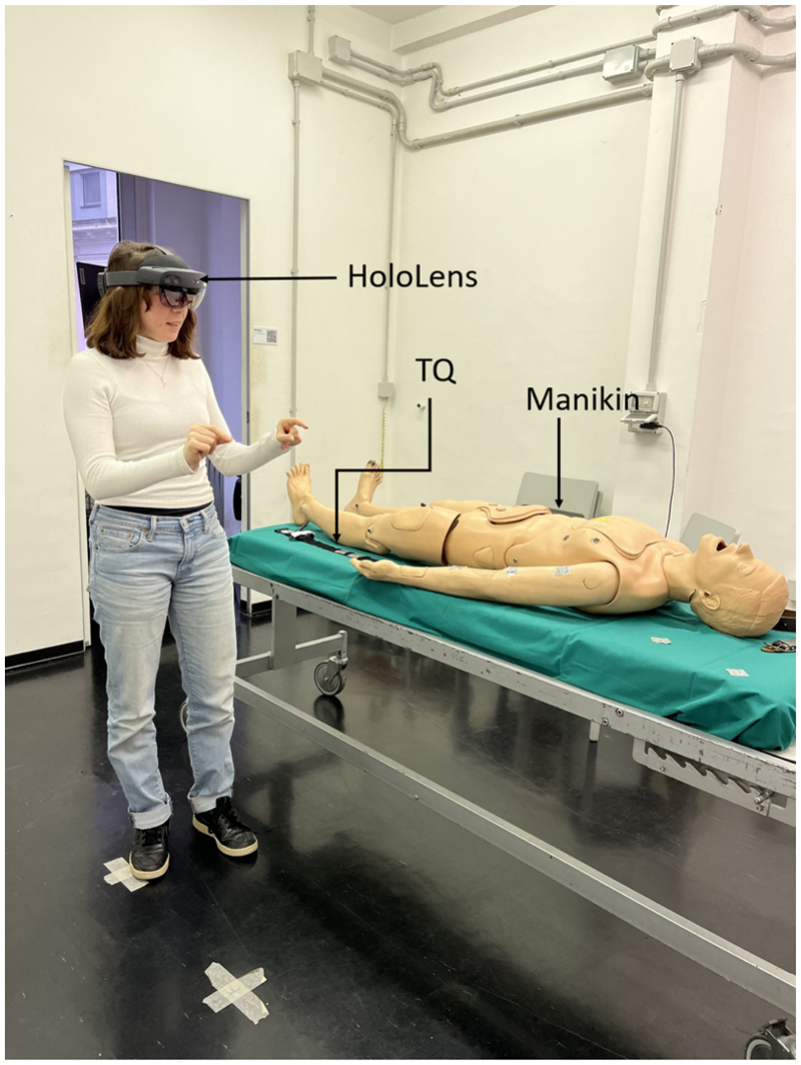
Components of the AR system: the Microsoft Hololens2 worn by the user, a manikin and a tourniquet (TQ).

The system combines a manikin, a Combat Application Tourniquet (CAT) tourniquet, and an AR application that leverages the HoloLens2 headset (Microsoft, USA) functionalities to guide unexperienced users in the application of a anti-hemorrhagic device (AHD) on a manikin’s limb (Figure 1). The HoloLens2 consists of sensors, cameras and a holographic processing unit to track the user’s environment and project holograms onto their field of view. This device allows for interaction with virtual holograms through gestures, voice commands and eye tracking.

The application we have developed put forth an emergency scenario to the user describing the manikin as a person experiencing limb bleeding. To handle the situation, the user is instructed with a set of tasks to help them using an anti-hemorrhagic device, which can be a tourniquet or an improvised one. Furthermore, the application provides a performance feedback in real time to guide the user toward a correct device positioning. We have decided not to include real-life conditions, such as the actual bleeding for two reasons: the end-user, namely a person without any medical background, and the skill to acquire. Indeed, the level of realism of a training should depend, among others, on the learners’ expertise and on the type of skill to be trained (32), as highly realistic simulations are more complex and impose a high cognitive load on the trainees (33).

### 3.1 AR application design and development

**Figure 2:**
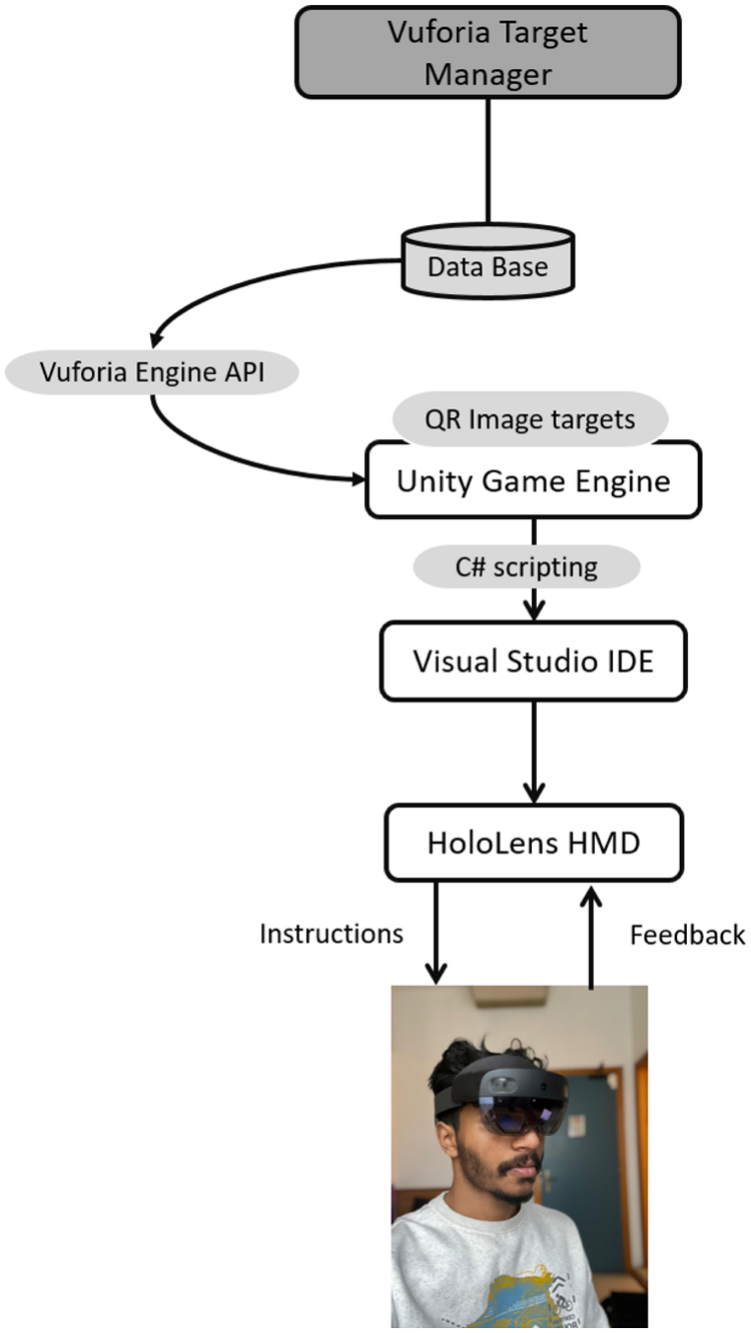
Main components of the proposed system: a data base of QR codes has been created and uploaded on Unity Game Engine. The Vuforia Engine APIs have been integrated with the Unity Engine to allow the device to detect and track the QR codes. The AR application has been written in C# and it is executed on the HoloLens2 device which allows for user’s interactions with the application.

The application is developed in Unity Game Engine version 2022.3.10f1, using C# scripting, and Visual Studio IDE for code compilation and deployment (Figure 2). The Mixed Reality Toolkit (MRTK) has been used to develop the AR contents. Additionally, the Vuforia Computer vision (V-CV) Software Development Kit has been used for implementing an interactive feedback mechanism. Indeed, the integration of the Vuforia Engine Application Programming Interfaces (APIs) into Unity Game Engine enables to use advanced computer vision algorithms, with the aim of implementing methodologies for image recognition and tracking, eventually allowing the application to recognize targets associated with interaction strategies to opportunely guide the user in the training process. In this sense, and to provide robust objects detection, QR codes have been used as image targets. A database with the used QR codes has been created; the same is uploaded into the Unity engine (Figure 2) in runtime, while the application continuously captures video frames. In parallel, the Vuforia APIs process these frames, enabling the Microsoft Hololens2 device to detect and track image targets.

With the aim of providing an opportune feedback to the user, different QR codes have been therefore placed on the physical parts of the system (i.e., manikin and tourniquet). Each QR codes create a sort of trigger zone, which is associated with a specific visual and vocal feedback. In other words, by using QR codes, the application can detect if a task has been correctly accomplished and provide a multimodal feedback, which allows the user to proceed with the next task, leaving no place for procedural errors to occur. The placement and the associated feedback of each QR code will be described more in details in the following sections.

**Figure 3:**
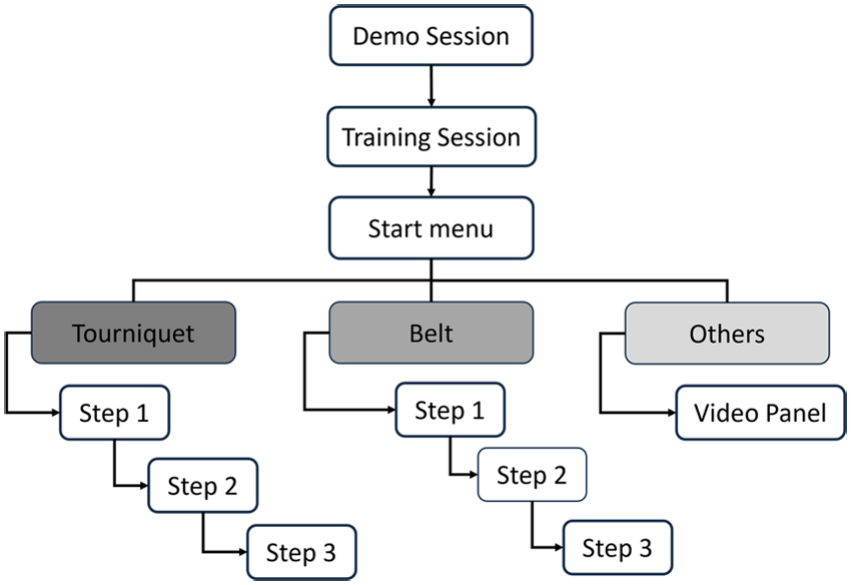
Workflow of the application: the application starts with a demo session. As soon as users feel ready to use the system, they can proceed with the training. Based on the choice of the anti-hemorrhagic device to use, the application will guide the user through different steps for placing it on the manikin’s limb Section 3.1.2.

The AR application that we have developed is divided into two main sessions: (i) the Demo session, designed to let the user familiarize with the technology, prior to the training; (ii) the Training (Simulation) session, to train the user on how to position an anti-hemorrhagic device. The overall workflow of the application is shown in Figure 3.

#### 3.1.1 Demo session

**Figure 4:**
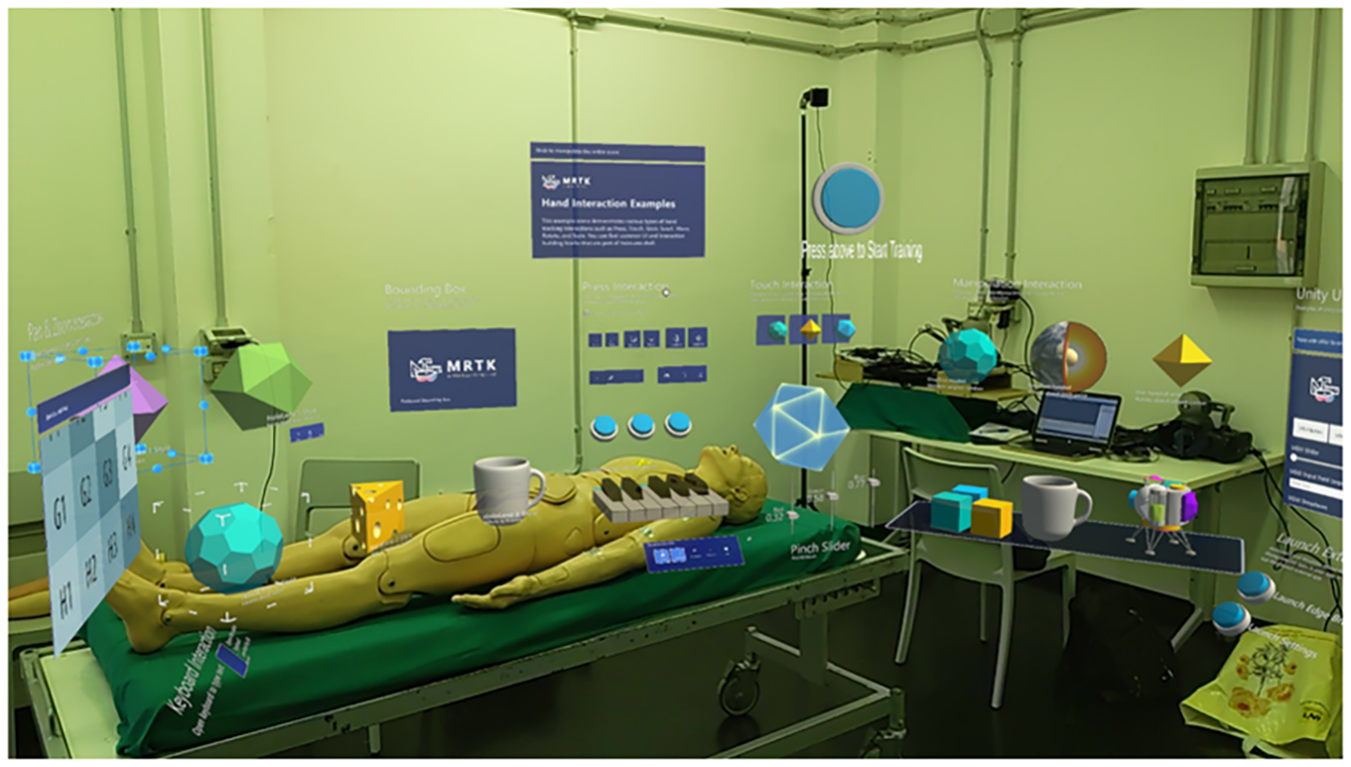
Demo session. It allows the user to get familiar with the application, and its interactions possibilities, such as grabbing and releasing virtual objects, QR codes, pressing virtual buttons.

The Demo session (Figure 4) has been implemented using the MRTK package and its hand tracking functionalities. This session has two main objectives: (i) to make the user familiar with the functionalities and possible interactions of the HoloLens2, providing a general understanding about how to handle objects and how to interact within the AR environment; (ii) to learn how to perform specific interactions that will be key during the training session. Indeed, virtual objects with QR codes have been added to the scene, so as to make the users aware of the minimum distance they need to be to enable the detection of the code (see also Supplementary Material).

The user is free to interact with the Demo session until they get comfortable in proceeding with the actual training session: an interactive button is placed in the application, so that, at any moment, the user can use the button to move to the training session (Figure 4).

#### 3.1.2 Training session

**Figure 5:**
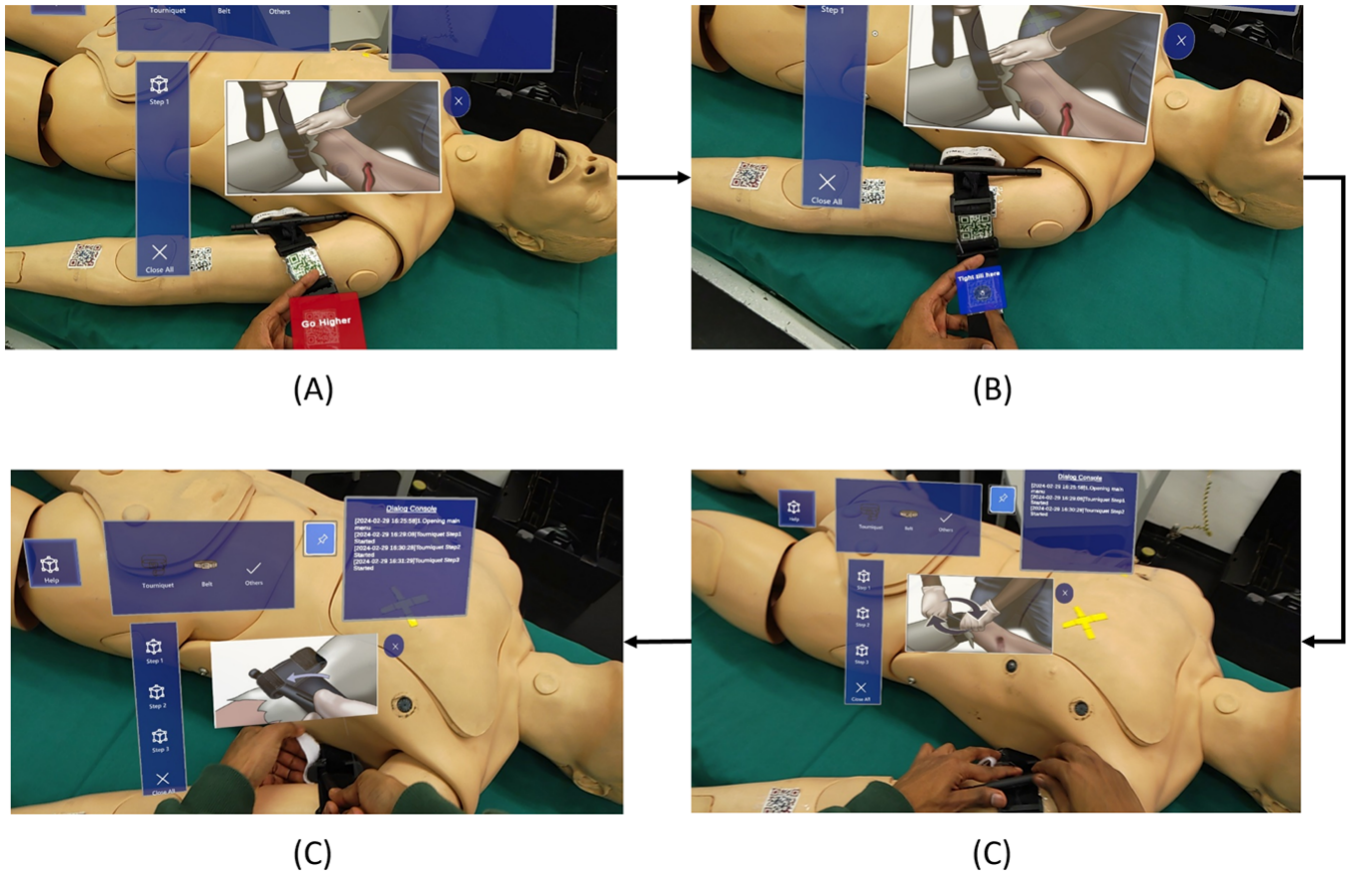
Snapshots of the application: **(A)** placement of the tourniquet in an incorrect position; **(B)** panel to confirm the correct tightening of the tourniquet; **(C)** instructions for twisting the windlass; **(D)** instructions for locking the windlass.

As the user ends the Demo session, the application starts the training scenario, and a User Interface Menu panel appears in the user’s field of view (Figure 5). This Panel provides feedback to the user throughout the training session. The application at first provides three options, i.e., instruments, that the user can choose to manage the hemorrhage: (i) Tourniquet; (ii) Belt; (iii) Others. Based on the user selection (i.e., users can touch the object they want to use), the application guides them on how to use and position the instrument, by using different types of feedback.

In the following, the main steps of the application for the different objects are described. See also the Supplementary Material for further screenshots of the application.

**Figure 6:**
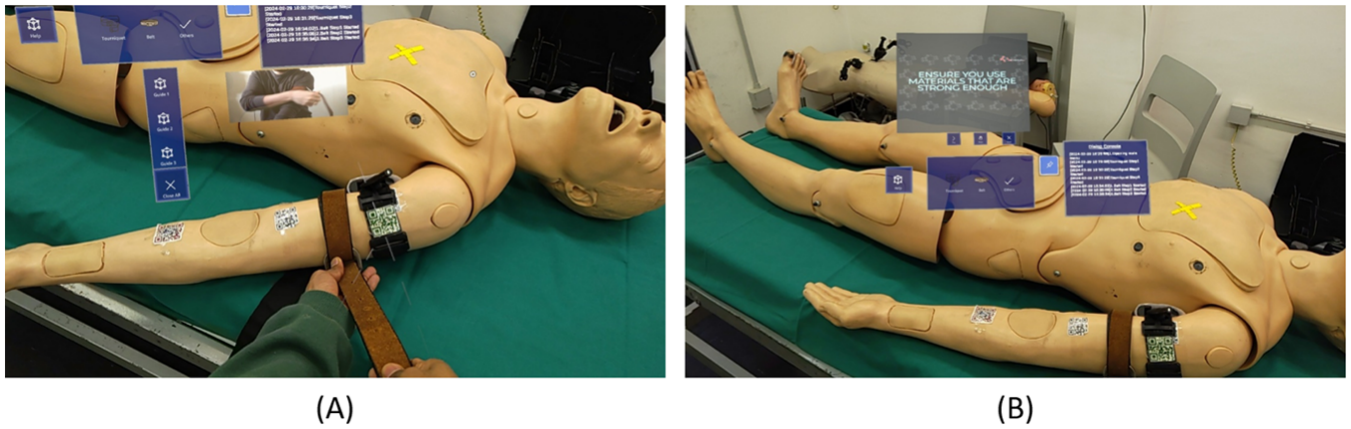
Snapshots of the application: Instructions on to use a belt as an anti-hemorrhagic device **(A)**; video panel providing practical instructions about how to create an improvised AHD **(B)**.

1. **Tourniquet**
  An audio cue instructs the users to take the tourniquet and guide them toward a correct positioning on the manikin’s arm. This is made by positioning QR codes on both the tourniquet and the arm, as mentioned in the beginning of the section. Concerning the arm, three different regions have been considered: a “correct region” (i.e., near the shoulder) and two “wrong regions”, and all of them are associated with a QR code (Figure 5). More in details, the application analyzes the relative position of the QR code associated to the tourniquet with respect to the three QR codes on the manikin arm, therefore identifying if the position of the instrument is correct or not, and it verbally notifies the user about the correct or wrong placement of the tourniquet. Eventually, only when the user successfully places the tourniquet in the correct area, a confirmation panel appears, enabling the user to move to the next step. The user can interact with the confirmation panel either verbally or by physically clicking the virtual button.
  Once the tourniquet is in the right position, the second step requires the user to properly tight the device (Figure 5). The application verbally and visually guides the user through the process; two QR markers have been attached to the tourniquet tightening area. One is placed below the actual tightening limit, so as to enable the detection of a loose fixing, while another is placed in correspondence of the proper level of tightening (Figure 5). As soon as the user complete the task, another panel pops up, so that the user can confirm their choice.
  Only if the tourniquet has been correctly tightened, the system proceeds with the third, final step, which consists in windlass twisting and locking. Also in this case, the application provides instructions to twist the windlass rod and lock it in the windlass clip, as shown in Figure 5C,D.
  At the completion of each step, the application also stores the related timestamps, giving the possibility to compute the time needed to perform each step, for subsequent performance analysis.
2. **Belt**
  If the user selects the belt as AHD, they are provided with audio and visual guides on how to use it as an improvised anti-hemorrhagic device. Specifically, a 3-button vertical panel shows up (Figure 6A), corresponding to three different steps and related instructions for using a belt in this context (provided, as for the tourniquet, in a multimodal manner). The three different steps consists in: (i) configuring the belt as an AHD, (ii) apply the belt in the right place, and (iii) tighten the belt.
3. **Others**
  If users select this option, on the initial menu, a video panel shows up with play, pause and close operations. By looking at the video as reference, the user can gain practical information on how to create, with a step by step approach, an improvised tourniquet with objects of common use, such as a piece of cloth and a wooden stick, or even with a pen (Figure 6B).

### 3.2 Experimental setup

To assess the usability of the developed system, we have tested the application on 20 subjects (age mean ± STD 27.05±4.2 years, 8 female) without any medical knowledge. Exclusion criteria were to be a student or graduate in a medical discipline (medicine, nursing, obstetrics, etc.; see Table 1 for further demographic details). Participants have been recruited by word of mouth. The study was approved by the local Institutional Review Board (code CE CERA protocol 2024/02 approved on 14/12/2023). To determine the sample size of our study, we have conducted an a priori power analysis using G*Power version 3.1 (34). Results indicated that the required sample size to achieve 80% power for detecting a medium effect, at a significance criterion of α=0.05, was N=15 for two tails paired T-test.

**Table 1:** Demographic data of the experiment’s participants.

ID	Gender	Age	Education	Field	VR/AR exp	Visual aid	Bleeding management exp
AR01	F	24	Ma	Engineering	VR, AR	G	/
AR02	M	41	Ba	Language	VR, AR	G	Yes
AR03	M	28	Ma	Engineering	/	/	/
AR04	M	23	Ba	Engineering	VR	G	/
AR05	M	27	Ma	Engineering	VR, AR	G	/
AR06	M	25	Ma	Engineering	VR	G	/
AR07	M	25	Ma	Engineering	/	G	/
AR08	M	25	Ba	Engineering	/	/	/
AR09	M	25	Ba	Engineering	VR, AR	/	Yes
AR10	M	28	Ma	Engineering	VR, AR	G	Yes
AR11	F	28	Ma	Engineering	/	/	/
AR12	F	26	Ma	Engineering	/	/	/
AR13	M	28	Ma	Engineering	VR	G	/
AR14	M	25	Ma	Engineering	VR	/	/
AR15	F	30	Ma	Engineering	VR	/	/
AR16	F	31	Ba	Economics	/	/	/
AR17	F	28	Ba	Communication	VR	/	/
AR18	M	19	Ba	Architecture	VR	/	/
AR19	F	26	Ba	Humanistic	VR	/	/
AR20	F	29	Ma	Biology	VR	G	/

Ma: Master’s degree; Ba: Bachelor Degree; G: glasses.

Participants started the experiment by filling out a demographic questionnaire, to collect information about gender, age, previous experience with AR, use of vision correction aids, and previous experience in bleeding management (Table 1), together with a Simulation Sickness Questionnaire [SSQ (35), refer to Section 3.2.1 for additional details]. Subsequently, each subject was given a briefing regarding how AR works and how the training was going to be carried out. In particular, general information about limb bleeding management and the tourniquet were provided, without however giving any insights about the usage of this device for bleeding management, since these aspects would have been part of the training session. The primary objective of the briefing was to make sure that all subjects could complete the experiment and knew the basic terminology related to bleeding management.

**Figure 7:**
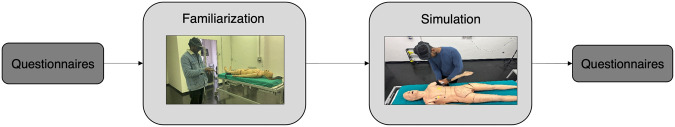
Experiment Pipeline. Subjects started the experiment by filling out a demographic questionnaire and SSQ. Then, they underwent a familiarization session, followed by a simulation of a massive limb bleeding. After the simulation, subjects filled out four questionnaires: SSQ, SUS, SIM-TLX, UEQ.

Prior to starting the simulation session, users wore the HoloLens2 and underwent a familiarization session, to understand how to use the device (i.e., how virtual objects are displayed and how to interact with them; see Section 3.1.1; Figure 7). Then, they started the training, which required managing an upper-limb massive hemorrhage with a tourniquet, by following the application’s training steps and feedback (see Section 3.1.2 for further details on the application). For each step (i.e., tourniquet positioning; tightening; twisting and locking the windlass), the application saves the time required to complete it. The proper tourniquet position was checked by the application, as the user could not proceed with the training until the tourniquet was positioned in the right place. Regarding the second and third steps, a single experimenter checked whether the participant successfully accomplished the task. After the simulation, participants filled out four questionnaires (Figure 7): (i) SSQ (35), to analyze and observe any discomfort caused by the AR application; (ii) System Usability Scale [SUS (36)]; (iii) User Experience Questionnaire [UEQ (37)]; (iv) Simulation Task Load Index [SIM-TLX (38)].

Nonparametric Mann Whitney test was used to determine significant performance differences between subjects, while one-sample Wilcoxon signed rank test was used to analyze questionnaires data. P values have been Bonferroni corrected for multiple comparison. Results were considered significant with a p value <0.05. All statistical analyses were conducted using JASP 0.19.

#### 3.2.1 Questionnaires

As mentioned before, we have selected different questionnaires to evaluate the AR application in terms of: simulator sickness; usability, namely the subjective experience resulting from the interaction with a MR system (39); workload which results from task demand, user’s behavior, skills and perception (38); subjective user experience.

**Simulation Sickness Questionnaire (SSQ)** is used for measuring users’ level of sickness symptoms, as a result of a MR simulation (40). This is a standardized questionnaire asking participant to rate 16 symptoms on a four-point scale (0–3). This questionnaire is presented to the subject twice: before and after the simulation, to ensure that any symptom that might occur is strictly related to the use of the AR application.

**System Usability Scale (SUS)** (36) is a standardized 10-item questionnaire with 5-points Likert scale; from strongly agree (5 points) to strongly disagree (1 point). Items cover three usability criteria: (i) effectiveness, namely the ability of participants to complete the tasks using the system; (ii) efficiency, the amount of mental resources needed to complete tasks; (iii) satisfaction, (i.e., the users’ subjective reactions to the system (39)). To analyze the data, the scores of the 10 questions need to be added and the resulting score should be multiplied by 2.5, to obtain a cent scale (41). Scores greater than 80.3 indicate an excellent usability, between 80.3 and 68 good usability; between 68 and 51 poor usability; scores lower than 51 insufficient usability (36).

**Simulation Task Load Index (SIM-TLX)** (38) is used to measure the workload experienced by the participants while performing tasks in simulation. This questionnaire assesses various dimensions of workload such as mental demands, physical demands, temporal demands, frustration, task complexity, situational stress, distraction, perceptual strain, task control and presence. For each dimension, participants rate the level of demand on a 21-point Likert scale anchored between low to high. 0 - being the lowest. 20 - being the highest. For each item, we have analyzed the mean and median value.

**User Experience Questionnaire (UEQ)** (37) includes 26 items assembled into 6 dependent scales: attractiveness, efficiency, perspicuity, dependability, originality, stimulation (42). Each UEQ item consists of a pair of terms with opposite meanings, and each item can be rated on a 7-point Likert scale from −3 (fully agreement with negative term) to +3 (fully agreement with positive term).

## 4 Results

All participants were able to complete the task, i.e., managed the upper limb hemorrhage, with the aid of the AR application. Demographic data revealed that most of the participants were unaware about common techniques to deal with massive hemorrhage management, with only three subjects who had basic previous knowledge (i.e., they heard about how to deal with a massive hemorrhage in a first aid course). Conversely, 14 subjects of 20 had used VR before the experiment and 5 of them were also familiar with AR (Table 1).

### 4.1 Performance

**Figure 8:**
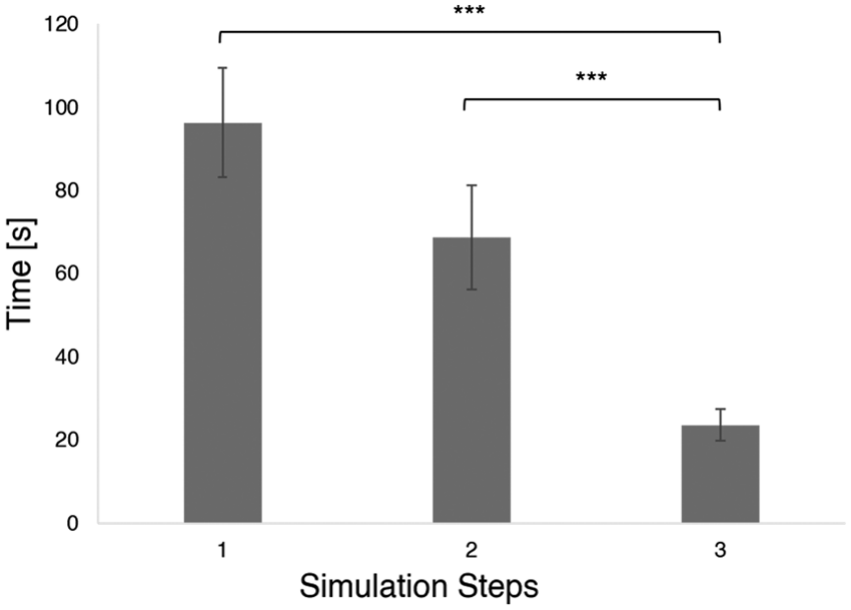
Mean and S.E. time required to complete each simulation step (i.e., step 1: tourniquet placement; step 2: tourniquet tightening; step 3: windlass lock) *** p<0.001.

As performance indicator, we have analyzed the time needed by all users to complete the demo, as well as those required to accomplish each step of the training session (see Section 3.1). Subjects spent on average 6 minutes to familiarize with the system, without differences between people with previous VR/AR experience (mean ± S.E.: 358±35 s) and people using an AR system for the first time (360±49 s; p=0.834). The time spent for the actual training was much less (240±26 s p=0.004). As expected, the first step (i.e., taking the tourniquet and position it in the right location), was the slowest (96±13 s), followed by the tightening of the device (step 2; 69±13 s) and the windlass twisting and locking (step 3; 24±4 s; Figure 8).

### 4.2 Questionnaires

**Figure 9:**
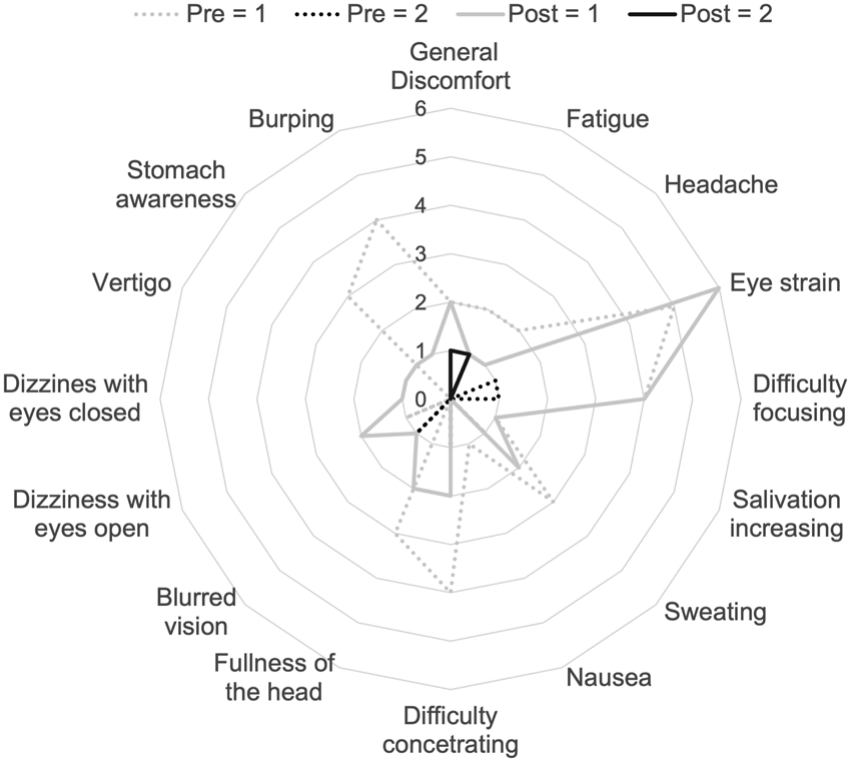
Number of subjects who indicated scores greater than 0 in the SSQ test. Dotted gray line: pre test values =1; dotted black line: pre test values =2; filled gray: Post test =1; filled black: post test =2. No subjects reported values greater than 2.

As one of the main drawbacks preventing the use of AR systems is the so-called Simulation Sickness, we have asked participants to rate their symptoms before and after the experiment, using SSQ (40). This way, we could establish whether the system would cause any symptom, or rather if the symptoms reported where related to the health status of the participants. Results show an overall low level of sickness, both before (mean across all the symptoms ± S.E. 0.14±0.08) and after the usage of the AR application (0.08±0.07; all p values greater than 0.05; Figure 9).

**Figure 10:**
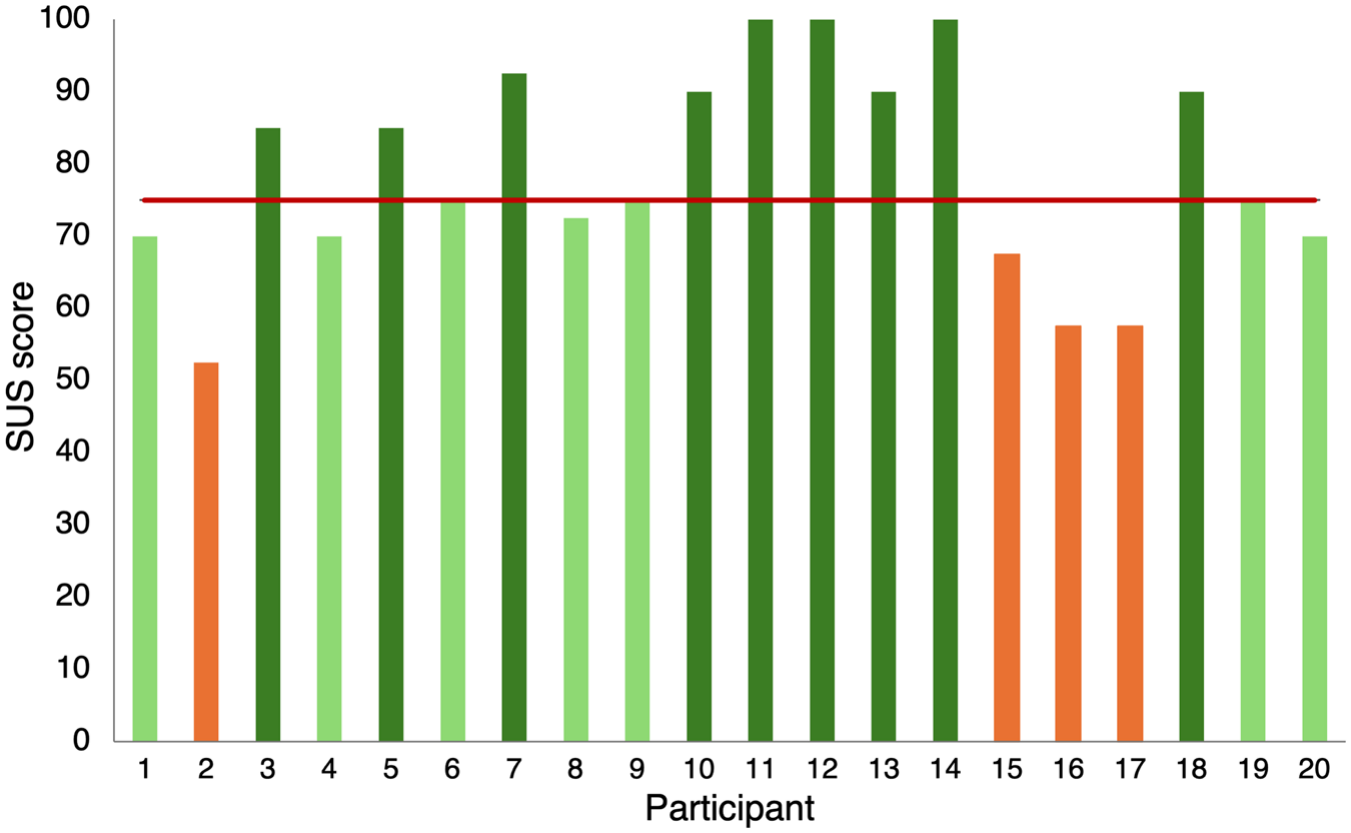
SUS scores. Each column represents the usability score provided by each participant. Colors indicates whether the usability was considered excellent (dark green), good (light green), (poor orange). The red bar shows the mean value.

Regarding System Usability (36), the mean of all the SUS scores obtained is 75 ± S.E. 3.2, which indicates an good usability score (p=0.006; Figure 10). Indeed, 9 out of the subjects provided a score greater than 80.3. None of the subjects reported a usability score lower than 51, and only 4 subjects rated the usability as poor (Figure 10).

**Figure 11:**
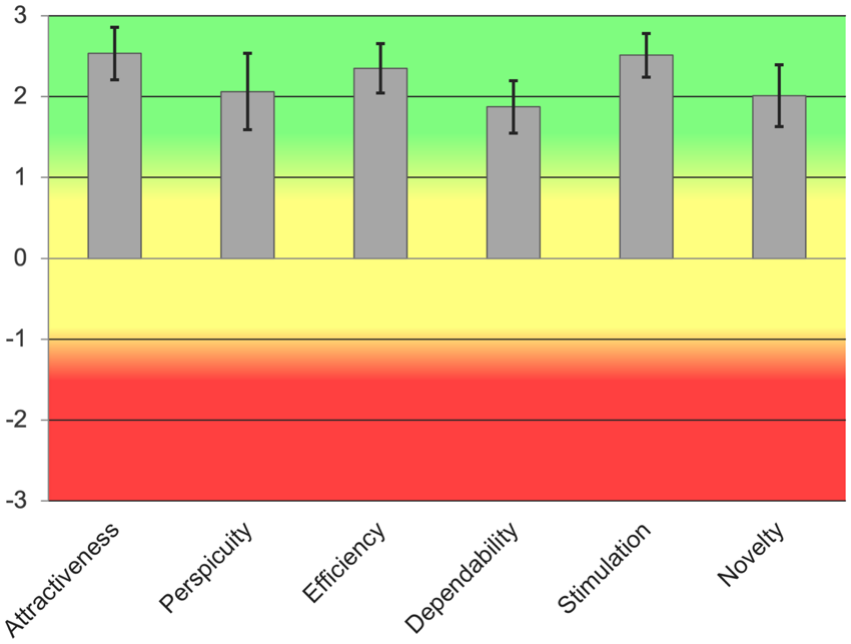
UEQ Scores mean and STD value for each scale. Green indicates good usability, yellow average usability, red low usability.

The results obtained from the UEQ (37) support the insights drawn from the SUS scores. Indeed, overall all the six items of the UEQ were rated positively by users (p<0.001 for all the items; mean ± S.E. across all the items: 2.2±0.3; Figure 11), confirming that the user experience was overall very good.

**Figure 12:**
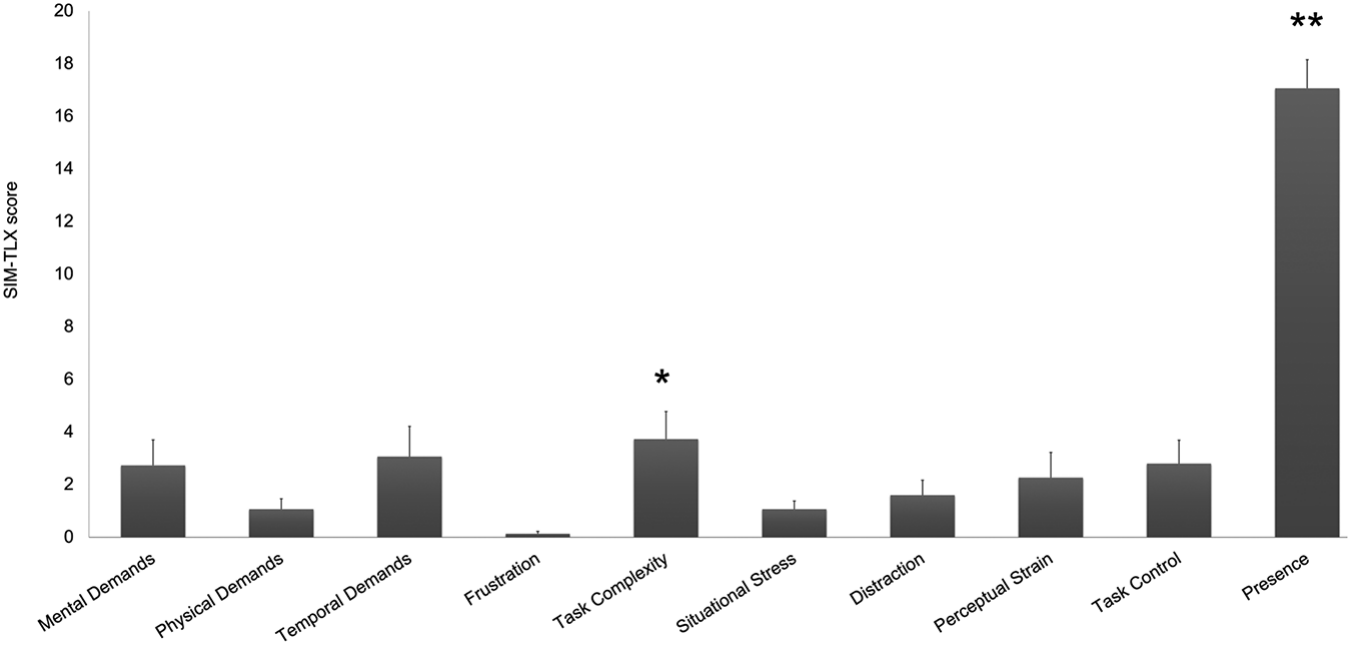
SIM-TLX Scores, mean and S.E. values for each dimension. *p≤0.05; **p≤0.01.

Finally, we asked the participants to fill out the SIM-TLX questionnaire (38) to assess whether performing the simulation with the AR system might cause any type of workload or fatigue. Results report an overall low workload resulting from the usage of the application. The only values which are significantly different than 0 are Task complexity (mean ± S.E. 3.7±1.1, p=0.04), and Presence (mean ± S.E. 17.1±1.1, p=0.01; Figure 12).

## 5 Discussion

Existing studies have identified the main elements facilitated by immersive technologies in: sensory realism, learner interaction, facilitator control and scenario immersion (43). The current work presents an AR application, based on the HoloLens2 device, which leverages two of these elements, namely, scenario immersion and learner interaction, to develop an application for training non-experienced users in the medical treating of upper limb hemorrhage. Even though the application moves along some predefined training steps, the duration of each step and the interaction modalities can be controlled by the user, allowing them to choose their feedback preference and to proceed at their own learning pace. Those positive aspects have been confirmed by a preliminary evaluation of the system done with 20 participants, which have effectively completed the AR training session, and positively evaluated the system in terms of usability and workload. By looking at the results, we can draw some interesting conclusions, whilst preliminary, about the proposed system:

• Although many participants had previous experience with VR/AR, the Demo/Familiarization session has been extensively used by all users, providing a sufficient level of expertise to carry out the training session. Hence, all the participants were able to complete the training in less than 5 min, thus suggesting that the familiarization session was effective to get acquainted with AR instruments. This result highlights the importance of designing a familiarization session prior to any VR/AR-based training experience, which let the user familiarize with the environment and its possible interactions. The importance of coupling VR/AR with a familiarization session is a highly debated topic in healthcare education, and more generally in all the fields where immersive applications are used by people without any technical knowledge and background. Indeed, a pre-training phase is desirable to avoid any negative outcomes that might be driven from the novelty effect (44). Further, providing users with a familiarization session would reduce intrinsic cognitive load (45), increase attention during the training (46), and release memory, and other cognitive resources, thus promoting learning while using immersive technologies (47).
• Users took between 1.5 to 5 min to properly position the tourniquet. Analysis on the single steps revealed that taking the tourniquet and position it was the slowest step, followed by tightening and locking it (Figure 8). One can argue that the application of a tourniquet for a massive bleed is a time-sensitive operation. In general, trained rescuers should be able to successfully apply a tourniquet in less than one minute (48, 49). However, the AR application that we developed was designed to teach untrained rescuers how to apply a tourniquet and not to support untrained lay rescuers in a real emergency. Therefore, it is reasonable to assume that a training would require more time than positioning a tourniquet in a real emergency. Future studies should assess whether the time required to apply a tourniquet decreases after the training with the AR application, suggesting that such system would be beneficial to the users in real emergencies. As one of the possibilities of AR is to provide immediate cues in real-life situations, it is crucial to acknowledge that performing an action with the aid of AR would usually take longer than without it. Therefore, research groups should focus on how to design applications that can be used in real time, especially in time sensitive situation, as the trauma and emergency medicine field. In this context, it might be useful to investigate the support of artificial intelligence to define the severity of hemorrhage and suggest the best way to the layperson to adopt to stop the bleeding.
• Another possible explanation of the fact that users took quite a long time to succeed with the tourniquet positioning might be that the cues provided were not optimal, and user might have struggled to complete the three steps, or interact with the AR application. As a matter of fact, users could interact in different ways with the application (i.e., with their hands, by completing actions, or verbally). These options have been implemented, as we wanted to explore the possibility of a personalized training application. In fact, one of the main differences between VR/AR-based applications and instructor-guided courses is that the former do not provide individualized learning (11). Studies investigating the impact of cues on learning and retention outcomes, reported that personalized cues promotes memory creation, access to mental states and strengthen memory accuracy and retention (50, 51). However, identifying optimal cues is a trial and error process which might slower the task accomplishment (52). Nevertheless, questionnaires results report an excellent system usability, a very good user experience and high values for immersion and sense of presence, thus suggesting that he AR interface was perceived as very usable, and that participants were able to quickly learn how to use the device and select the most appropriate cue.
• Regarding the questionnaires results, that allowed us to collect information about the subjective experience while using the application, we can conclude that, other than being usable, the AR application has neither caused sickness symptoms, nor workload on users. Simulation sickness and workload are two crucial aspects to evaluate when developing a VR/AR application, as they often affect the usage of such applications, as well as its training potentialities. Simulation sickness is a frequent phenomenon occurring when people use VR/AR (53); even though many theories have been developed to explain its incidence, it is still unclear which factors can cause such discomfort, and how to mitigate it (54). Furthermore, simulation sickness has been extensively studied in VR, while its occurrence in AR is still debated (55). With out results we can conclude that our AR application does not provoke any simulation-related symptoms. Studies on workload instead positively related performance (56) with mental workload. Further, cognitive workload resulting from using AR seems greater than traditional training methods (57). In our study, the reported workload is overall very low (Figure 12), despite all the subjects succeeded in performing the task. Future studies should investigate whether (i) massive hemorrhage training using the AR would be more effective and demanding than traditional methods; (ii) multimodal cues affects mental workload.
• Our result is in line with other usability studies on AR/VR for medical training. In fact, people testing VR/AR tools, either experienced healthcare providers or untrained learners, rate them generally positively in terms of user experience and usability (58, 59). As reported by (60), hand interactions might be challenging and therefore require some practice to master them. Some of our subjects had the same issue while using the AR application; this has been reflected on a slightly lower values for the user experience questionnaire item “dependability”, that is the impression to be in contact with the product, with respect to other items. A study from 2017 which investigated whether non-verbal cues could improve VR-based first aid training, indicated that icons can be used for the training of people without medical knowledge (61). The comparison of the present results with similar usability studies suggests the proposed application can be beneficial for medical learning, without affecting user experience and usability.
• As said above, during the experiment, some subjects found it challenging to properly interact with the system; particularly, they took some time on understand how to press buttons and handle virtual objects. Even though these difficulties have not affected the overall system’s usability, the task complexity was significantly greater than zero. The future version of the AR application should simplify the interactions, and/or offer alternatives on how to control virtual objects (e.g., by pointing toward a virtual button to press it, or touching it; grasping a virtual object with the entire hand or pinch it with two fingers). Moreover, some subjects particularly appreciated the audio cues. In view of this, future development includes the implementation of conversational agents and interactive virtual assistants that would provide tailored advice and allow for a verbal user-system communication. Altogether, the above-mentioned improvements heads towards an increasingly personalized training.

### 5.1 Limitations

For this study, we have enrolled subjects who did not have medical background, as the target population would be people without medical knowledge who need to be trained in limb hemorrhage control. However, most of the participants had an engineering background, which could potentially lead to biases in the results. Indeed, people with a technical background could be more technologically proficient than people with a nontechnical education. That said, among them there were robotics and biomedical engineers, but also environmental, naval, and industrial engineers having very different skills and interests. In addition, participants ranged from first-year bachelor students up to graduated people currently working in various fields. System’s usability results seem not to be affected by either the educational background or the previous exposure to VR/AR. Indeed, out of the 4 subjects who rated the usability as poor, one had an engineering background, but three had experience with VR/AR.

Another limitation concerns the number of subjects enrolled for this study. Even though the power analysis suggests that 20 subjects are enough to assess system’s usability and workload, the sample might be too small to generalize the findings. In this regard, international and multi centric studies will be advisable.

Finally, the present study does not evaluate skills’ learning and retention and does not compare the proposed system with traditional training methods (i.e., videos, classes or manikin-based simulation). Indeed, we focused on the development and presentation of an AR application for limb massive hemorrhage management training that combines AR with multimodal cues. Results show that all the users successfully completed the task, suggesting that the application will let people acquire a skill. However, future studies are needed to (i) confirm this assumption; (ii) assess whether this skill is retained and for how long; (iii) compare the learning efficacy with other training methods traditionally used to train people how to position an anti-hemorrhagic device.

To the best of our knowledge, the proposed application represents the first system which combines AR with multimodal cues for the training of anti-hemorrhagic device positioning. Although no direct measures have been done yet on the effectiveness of the training, those preliminary results suggest that the proposed application could be an effective strategy to train inexperienced users to use an anti-hemorrhage device, being perceived as engaging, easy to use, and not demanding. Future tests will be aimed at exploring the effectiveness of such a training, assessing if participants are able to transfer the acquired skills in a real scenario. Also, this technology can be promising to expand the number of trained citizen in hemorrhage control reducing the number of instructors needed.

## Data Availability

The raw data supporting the conclusions of this article will be made available by the authors, without undue reservation.

## References

[1] Eastridge B Holcomb J Shackelford S. Outcomes of traumatic hemorrhagic shock and the epidemiology of preventable death from injury. Transfusion. (2019) 59:1423–8. 10.1111/trf.15161 30980749

[2] Petrone P Baltazar G Jacquez RA Akerman M Brathwaite CE Joseph DK. Stop the bleed: a prospective evaluation and comparison of tourniquet application in security personnel versus civilian population. Am Surg^TM^. (2023) 89:2481–5. 10.1177/00031348221101489 35567282

[3] Picard C. Hemorrhage control, a fundamental skill: a review of direct pressure, dressings, wound packing and bandages for life-saving. Can J Emerg Nurs. (2020) 40:26–8. 10.29173/cjen76

[4] Latina R Iacorossi L Fauci A Biffi A Castellini G Coclite D, Effectiveness of pre-hospital tourniquet in emergency patients with major trauma and uncontrolled haemorrhage: a systematic review and meta-analysis. Int J Environ Res Public Health. (2021) 18:12861. 10.3390/ijerph182312861 34886586 PMC8657739

[5] Smith A Ochoa J Wong S Beatty S Elder J Guidry C, et al. Prehospital tourniquet use in penetrating extremity trauma: Decreased blood transfusions and limb complications. J Trauma Acute Care Surg. (2018) 86:1. 10.1097/TA.0000000000002095 30358768

[6] Richey SL. Tourniquets for the control of traumatic hemorrhage: a review of the literature. World J Emerg Surg WJES. (2007) 2:28–. 10.1186/1749-7922-2-28 17958899 PMC2151059

[7] Zideman DA Singletary EM Borra V Cassan P Cimpoesu CD De Buck E, et al. European resuscitation council guidelines 2021: first aid. Resuscitation. (2021) 161:270–90. 10.1016/j.resuscitation.2021.02.013 33773828

[8] Lundberg M. *Error identification in tourniquet use: error analysis of tourniquet use in trained and untrained populations* (2020) 90

[9] ACS. Stop the bleed (2023). Available online at: https://www.stopthebleed.org/ (accessed June 30, 2024)

[10] Demaria S Levine A Sim A Schwartz A. The Comprehensive Textbook of Simulation in Healthcare. New York, NY: Springer (2013). 10.1007/978-1-4614-5993-4

[11] Ricci S Calandrino A Borgonovo G Chirico M Casadio M. Viewpoint: Virtual and augmented reality in basic and advanced life support training. JMIR Serious Games. (2022) 10:e28595. 10.2196/28595 35319477 PMC8987970

[12] Kobayashi L Zhang X Collins S Karim N Merck D. Exploratory application of augmented reality/mixed reality devices for acute care procedure training. Western J Emerg Med. (2018) 19:158–64. 10.5811/westjem.2017.10.35026 PMC5785186 29383074

[13] Tang Y Chau KY Kwok A Zhu T Ma X. A systematic review of immersive technology applications for medical practice and education - trends, application areas, recipients, teaching contents, evaluation methods, and performance. Educ Res Rev. (2022) 35:100429. 10.1016/j.edurev.2021.100429

[14] Jacobs C Foote G Joiner R Williams M. A narrative review of immersive technology enhanced learning in healthcare education. Int Med Educ. (2022) 1:43–72. 10.3390/ime1020008

[15] Iqbal AI Aamir A Hammad A Hafsa H Basit A Oduoye MO, et al. Immersive technologies in healthcare: an in-depth exploration of virtual reality and augmented reality in enhancing patient care, medical education, and training paradigms. J Primary Care Commun Health. (2024) 15:21501319241293311. 10.1177/21501319241293311 PMC11528804 39439304

[16] Tang KS Cheng DL Mi E Greenberg PB. Augmented reality in medical education: a systematic review. Can Med Educ J. (2020) 11:e81–e96. 10.36834/cmej.61705 32215146 PMC7082471

[17] Dhar P Rocks T Samarasinghe RM Stephenson G Smith C. Augmented reality in medical education: students’ experiences and learning outcomes. Med Educ Online. (2021) 26:1953953. 10.1080/10872981.2021.1953953 34259122 PMC8281102

[18] Huang X Yan Z Gong C Zhou Z Xu H Qin C, et al. A mixed-reality stimulator for lumbar puncture training: a pilot study. BMC Med Educ. (2023) 23:178. 10.1186/s12909-023-04173-9 36949483 PMC10035206

[19] Chytas D Johnson E Piagkou M Mazarakis A Babis G Chronopoulos E, et al. The role of augmented reality in anatomical education: an overview. Ann Anat Anatomischer Anzeiger. (2020) 229:151463. 10.1016/j.aanat.2020.151463 31978568

[20] Cercenelli L De Stefano A Billi AM Ruggeri A Marcelli E Marchetti C, et al. AEducaAR, anatomical education in augmented reality: a pilot experience of an innovative educational tool combining AR technology and 3D printing. Int J Environ Res Public Health. (2022) 19:1024. 10.3390/ijerph19031024 35162049 PMC8834017

[21] Dubron K Verbist M Jacobs R Olszewski R Shaheen E Willaert R. Augmented and virtual reality for preoperative trauma planning, focusing on orbital reconstructions: a systematic review. J Clin Med. (2023) 12:5203. 10.3390/jcm12165203 37629251 PMC10455745

[22] Aung S Punsawad Y. Application of augmented reality technology for chest ECG electrode placement practice. Informatics. (2024) 11. 10.3390/informatics11010005

[23] Balian S McGovern S Abella B Blewer A Leary M. Feasibility of an augmented reality cardiopulmonary resuscitation training system for health care providers. Heliyon. (2019) 5:e02205. 10.1016/j.heliyon.2019.e02205 31406943 PMC6684477

[24] Leary M McGovern S Balian S Abella B Blewer A. A pilot study of CPR quality comparing an augmented reality application vs. a standard audio-visual feedback manikin. Front Digit Health. (2020) 2:1. 10.3389/fdgth.2020.00001 34713015 PMC8521903

[25] Strada F Bottino A Lamberti F Mormando G Ingrassia PL. Holo-BLSD – a holographic tool for self-training and self-evaluation of emergency response skills. IEEE Trans Emerg Top Comput. (2019) PP:1. 10.1109/TETC.2019.2925777

[26] Drummond D Arnaud C Guedj R Duguet A Suremain N Petit A. Google glass for residents dealing with pediatric cardiopulmonary arrest: a randomized, controlled, simulation-based study. Pediatr Crit Care Med. (2016) 18:1. 10.1097/PCC.0000000000000977 28165347

[27] Park JC Kwon HJE Chung CW. Innovative digital tools for new trends in teaching and assessment methods in medical and dental education. J Educ Eval Health Prof. (2021) 18:13. 10.3352/jeehp.2021.18.13 34182619 PMC8376582

[28] Palumbo A. Microsoft HoloLens 2 in medical and healthcare context: State of the art and future prospects. Sensors. (2022) 22:1–30. 10.3390/s22207709 36298059 PMC9611914

[29] Maleszka L Breuer-Kaiser A Schäfer M Lehn K Felderhoff T. Proof-of-concept: conceptual design and realisation of an educational augmented reality (AR) application for anaesthesia induction. Curr Direct Biomed Eng. (2023) 9:250–3. 10.1515/cdbme-2023-1063

[30] Schulz F Nguyen Q Baetzner A Schrom-Feiertag H Gyllencreutz L. Mixed reality–exploring the requirements of realism in the context of mass casualty incident training. Prehosp Disaster Med. (2023) 38:s31. 10.1017/S1049023X2300119X

[31] Koutitas G Smith S Lawrence G. Performance evaluation of AR/VR training technologies for EMS first responders. Virtual Real. (2021) 25:83–94. 10.1007/s10055-020-00436-8

[32] Wilson E Hewett DG Jolly BC Janssens S Beckmann MM. Is that realistic? the development of a realism assessment questionnaire and its application in appraising three simulators for a gynaecology procedure. Advances in Simulation. (2018) 3:1–7. 10.1186/s41077-018-0080-7 30455991 PMC6225559

[33] Svendsen MBS Achiam MP. Defining medical simulators for simulation-based education in EUS: theoretical approach and a narrative review. Endosc Ultrasound. (2022) 11:95–103. 10.4103/EUS-D-21-00123 35488621 PMC9059801

[34] Faul F Erdfelder E Lang AG Buchner A. G*power 3.1.7: a flexible statistical power analysis program for the social, behavioral and biomedical sciences. Behav Res Methods. (2013) 39:175–91. 10.3758/BF03193146 17695343

[35] Kennedy RS Lane NE Berbaum KS Lilienthal MG. Simulator sickness questionnaire: an enhanced method for quantifying simulator sickness. Int J Aviat Psychol. (1993) 3:203–20. 10.1207/s15327108ijap0303_3

[36] Brooke J. SUS – a quick and dirty usability scale (1996) 189–194

[37] Laugwitz B Held T Schrepp M. Construction and evaluation of a user experience questionnaire. USAB 2008. (2008) 5298:63–76. 10.1007/978-3-540-89350-9_6

[38] Harris D Wilson M Vine S. Development and validation of a simulation workload measure: the simulation task load index (SIM-TLX). Virtual Real. (2020) 24:1–10. 10.1007/s10055-019-00422-9

[39] Borsci S Federici S Lauriola M. On the dimensionality of the system usability scale: a test of alternative measurement models. Cogn Process. (2009) 10:193–7. 10.1007/s10339-009-0268-9 19565283

[40] Bimberg P Weissker T Kulik A. On the usage of the simulator sickness questionnaire for virtual reality research (2020) 464–467. 10.1109/VRW50115.2020.00098

[41] Grier R Bangor A Kortum P Peres S. The system usability scale. Proc Hum Fact Ergon Soc Annu Meeting. (2013) 57:187–91. 10.1177/1541931213571042

[42] Schrepp M Hinderks A Thomaschewski J. Construction of a benchmark for the user experience questionnaire (UEQ). Int J Interact Multimed Artif Intell. (2017) 4:40–4. 10.9781/ijimai.2017.445

[43] Barrie M Socha J Mansour L Patterson E. Mixed reality in medical education: a narrative literature review. Proc Int Symp Hum Fact Ergon Health Care. (2019) 8:28–32. 10.1177/2327857919081006

[44] Miguel-Alonso I Checa D Guillen-Sanz H Bustillo A. Evaluation of the novelty effect in immersive virtual reality learning experiences. Virtual Real. (2024) 28:27. 10.1007/s10055-023-00926-5

[45] Meyer OA Omdahl MK Makransky G. Investigating the effect of pre-training when learning through immersive virtual reality and video: a media and methods experiment. Comput Educ. (2019) 140:103603. 10.1016/j.compedu.2019.103603

[46] Yong J Wei J Wang Y Dang J Lei X Lu W. Heterogeneity in extended reality influences procedural knowledge gain and operation training. IEEE Trans Learn Technol. (2023) 16:1014–33. 10.1109/TLT.2023.3286612

[47] Pollard KA Oiknine AH Files BT Sinatra AM Patton D Ericson M, et al. Level of immersion affects spatial learning in virtual environments: results of a three-condition within-subjects study with long intersession intervals. Virtual Real. (2020) 24:783–96. 10.1007/s10055-019-00411-y

[48] Shackelford SA Butler FK Kragh JF Stevens RA Seery JM Parsons DL, et al. Optimizing the use of limb tourniquets in tactical combat casualty care: TCCC guidelines change 14-02. J Spec Oper Med. (2015) 15:17–31. 10.55460/TDTK-RIN8 25770795

[49] Heldenberg E Aharony S Wolf T Vishne T. Evaluating new types of tourniquets by the Israeli Naval special warfare unit. Disaster Mil Med. (2015) 1:1–7. 10.1186/2054-314X-1-1 28265416 PMC5327874

[50] Tullis JG Finley JR. Self-generated memory cues: effective tools for learning, training, and remembering. Policy Insights Behav Brain Sci. (2018) 5:179–86. 10.1177/2372732218788092

[51] Noushad B Van Gerven PW de Bruin AB. Exploring the use of metacognitive monitoring cues following a diagram completion intervention. Adv Health Sci Educ. (2024) 29(4):1323–51. 10.1007/s10459-023-10309-9 PMC11368990 38285312

[52] Stawarz K Gardner B Cox A Blandford A. What influences the selection of contextual cues when starting a new routine behaviour? an exploratory study. BMC Psychol. (2020) 8:1–11. 10.1186/s40359-020-0394-9 32228721 PMC7106637

[53] Firdaus R Tantri AR Manggala SK. Factors influencing virtual reality sickness in emergency simulation training. Med Sci Educ. (2024) 1:1–7. 10.1007/s40670-024-02102-z PMC11699192 39758480

[54] Dużmańska N Strojny P Strojny A. Can simulator sickness be avoided? a review on temporal aspects of simulator sickness. Front Psychol. (2018) 9:2132. 10.3389/fpsyg.2018.02132 30459688 PMC6232264

[55] Kaufeld M Mundt M Forst S Hecht H. Optical see-through augmented reality can induce severe motion sickness. Displays. (2022) 74:102283. 10.1016/j.displa.2022.102283

[56] Jeffri NFS Rambli DRA. A review of augmented reality systems and their effects on mental workload and task performance. Heliyon. (2021) 7:e06277. 10.1016/j.heliyon.2021.e06277 33748449 PMC7969906

[57] Alessa FM Alhaag MH Al-Harkan IM Ramadan MZ Alqahtani FM. A neurophysiological evaluation of cognitive load during augmented reality interactions in various industrial maintenance and assembly tasks. Sensors. (2023) 23:7698. 10.3390/s23187698 37765755 PMC10536580

[58] Wong MAME Chue S Jong M Benny HWK Zary N. Clinical instructors’ perceptions of virtual reality in health professionals’ cardiopulmonary resuscitation education. SAGE Open Med. (2018) 6:2050312118799602. 10.1177/2050312118799602 30245815 PMC6144504

[59] Pedram S Kennedy G Sanzone S. Assessing the validity of vr as a training tool for medical students. Virtual Real. (2024) 28:15. 10.1007/s10055-023-00912-x

[60] Ingrassia PL Mormando G Giudici E Strada F Carfagna F Lamberti F, et al. Augmented reality learning environment for basic life support and defibrillation training: usability study. J Med Internet Res. (2020) 22:e14910. 10.2196/14910 32396128 PMC7251481

[61] Blome T Diefenbach A Rudolph S Bucher K von Mammen S. VReanimate—non-verbal guidance and learning in virtual reality. In: 2017 9th International Conference on Virtual Worlds and Games for Serious Applications (VS-Games). IEEE (2017). p. 23–30.

