# Supplementary material for: Augmented Reality for extremity hemorrhage training: a usability study
Source: Front Digit Health. 2025 Jan 6;6:1479544. doi: 10.3389/fdgth.2024.1479544 (PMC11743514; doi:10.3389/fdgth.2024.1479544)
Supplement: Supplementary file 1 [file Datasheet1.docx]

Supplementary Materials


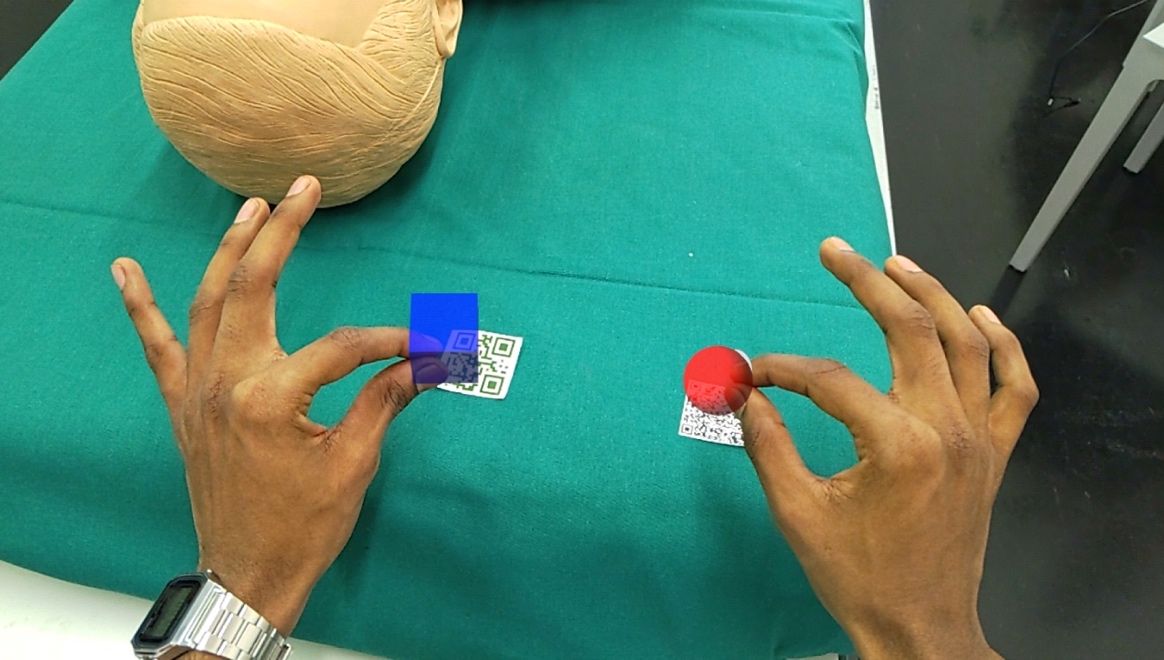


**Supplementary Figure 1.** Familiarization Session. Practice with QR marker detection using Vuforia Engine API. This helps users to understand the minimum threshold they need to focus on the marker for them to detect the markers.


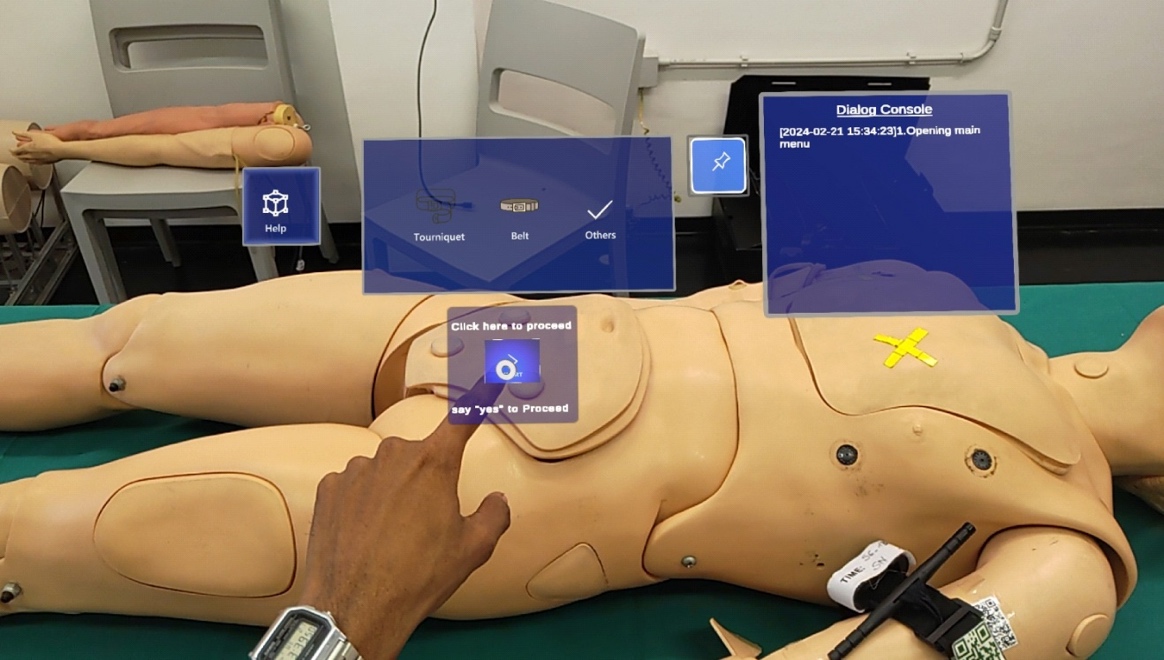


**Supplementary Figure 2.** Simulation session. User Interface panel visible during the simulation.


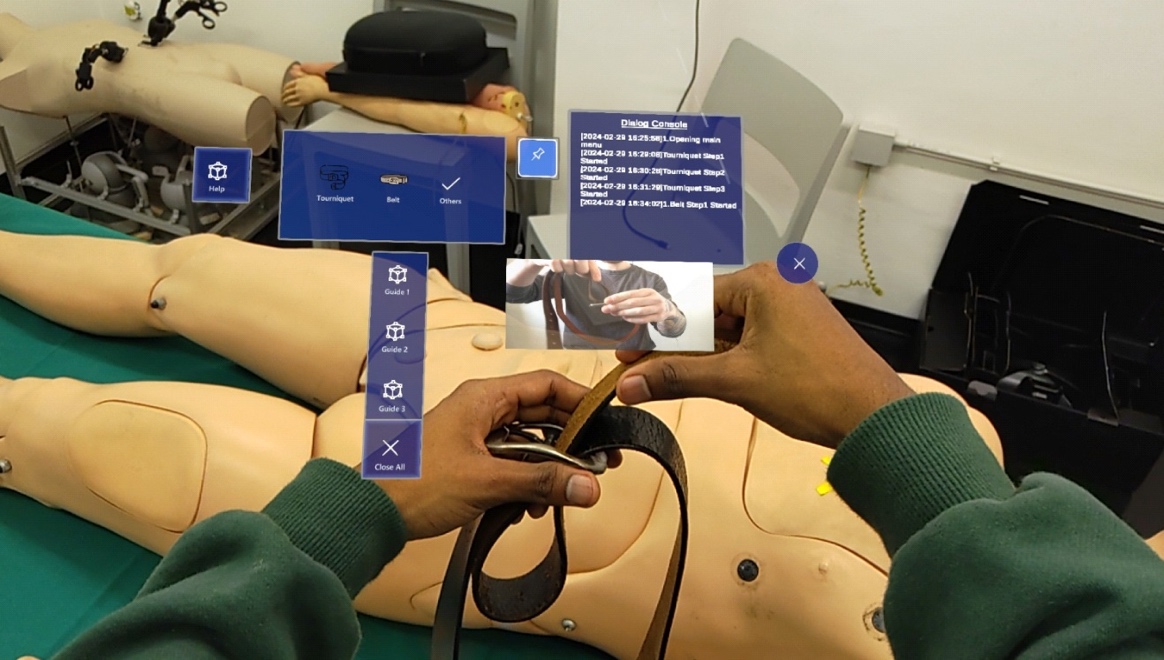


**Supplementary Figure 3.** Simulation session. Instructions on how to use a belt as an anti-hemorrhagic device, with Picture references, step 1.


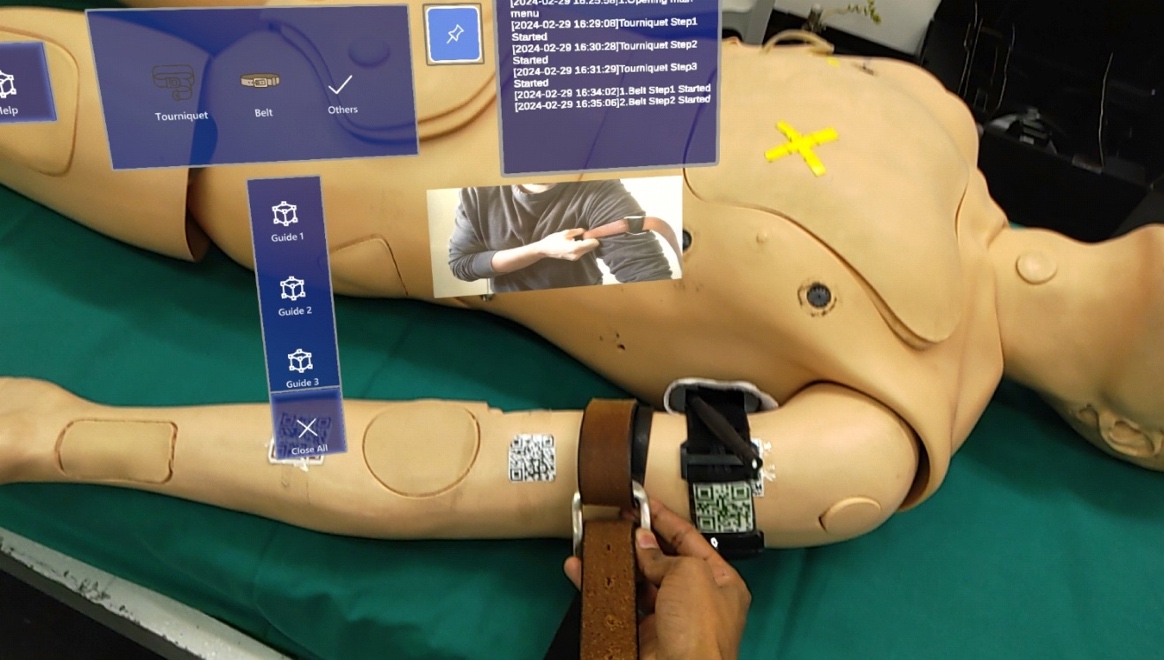


**Supplementary Figure 4.** Simulation session. Instructions on how to use a belt as an anti-hemorrhagic device, with Picture references, step 2.

A video showing the behavior of the application is available at the following link: <https://www.youtube.com/watch?v=sL9jPt96UHI&feature=youtu.be>
